# Perceived Stress Positively Relates to Insomnia Symptoms: The Moderation of Resilience in Chinese Pregnant Women During COVID-19

**DOI:** 10.3389/fpsyt.2022.856627

**Published:** 2022-04-27

**Authors:** Hongyu Zou, Zhen Tao, Yongjie Zhou, Zhiguo Zhang, Chunyan Zhang, Linling Li, Jiezhi Yang, Yanni Wang, Wei Huang, Jianhong Wang

**Affiliations:** ^1^Shenzhen Mental Health Center/Shenzhen Kangning Hospital, Shenzhen, China; ^2^School of Psychology, South China Normal University, Guangzhou, China; ^3^School of Biomedical Engineering, Health Science Center, Shenzhen University, Shenzhen, China; ^4^Shenzhen Health Development Research Center, Shenzhen, China; ^5^Department of Maternal, Child and Adolescent Health, School of Public Health, Lanzhou University, Lanzhou, China

**Keywords:** perceived stress, sleep quality, resilience, pregnant women, moderation effect

## Abstract

**Background:**

The government’s COVID-19 pandemic response lockdown strategy had a negative psychological and physical impact on individuals, which necessitated special care to pregnant women’s mental health. There has been no large-scale research on the underlying relationship between perceived stress and insomnia symptoms in pregnant Chinese women up to this point. During the COVID-19 pandemic, we wanted to see if there was an association between perceived stress and insomnia symptoms, as well as the moderating impact of resilience for Chinese pregnant women.

**Methods:**

This cross-sectional study examined 2115 pregnant women from central and western China using multi-stage sampling methodologies. A systematic questionnaire was used to collect information on sleep quality, perceived stress, and resilience using the Insomnia Severity Index, Perceptual Stress Scale, and Connor and Davidson Resilience Scale. To assess the moderating influence of resilience, hierarchical regressions were used.

**Results:**

During the COVID-19 pandemic, 18.53% of respondents (*N* = 2115) reported experiencing sleeplessness. In pregnant women, perceived stress was positively linked with insomnia symptoms (*p* < 0.001). Furthermore, resilience significantly attenuated the influence of perceived stress on insomnia symptoms in Chinese expectant mother (β_interaction_ = −0.0126, *p* < 0.001).

**Conclusion:**

Pregnant women with strong resilience were less influenced by perceived stress than those with poor resilience. The findings of this study might give empirical proof that health care professionals should identify the relevance of reducing perceived stress in pregnant women with poor resilience and provide better treatment and support when necessary.

## Highlights

- During the COVID-19 pandemic, according to the present study 18.53% of 2,115 pregnant women are having sleeplessness.
- We evaluated the influence of pregnant women’s perceived stress and resilience on sleep quality during the fast spread of the COVID-19 pandemic.
- Resilience were negatively correlated with perceived stress and insomnia severity, while perceived stress was positively correlated with insomnia severity, indicating that higher perceived stress was associated with lower resilience and higher insomnia severity, whereas higher resilience was associated with lower insomnia severity.
- Resilience during COVID-19 may moderate the relationship between stress and insomnia symptoms, paving the way for future mental health treatments in public health emergencies.

## Introduction

COVID-19 made its debut in December of 2019. It has resulted in a worldwide health catastrophe that is catastrophic. By the end of June 2021, there had been more than 17,277,958 confirmed cases globally, resulting in 3,711,711 fatalities. Coronavirus infection not only poses a serious threat to one’s physical well-being, but it also has a number of long-term consequences, such as cognitive impairment, persistent tiredness, and discomfort (1). As a result, its influence on mental health, such as the high frequency of stress, anxiety, and depression, has been widely discussed (2). Researchers are paying increasing attention to the influence of social isolation under the Chinese government’s home isolation policy to contain the spread of the virus. A related study has looked at the mental health of children who have been subjected to quarantine (3) and found that it is crucial to address the increasing anxiety and depressive symptoms in youngsters (4). Admittedly, only a few studies have focused on the insomnia symptoms of pregnant women during the pandemic in china’s context (5, 6).

Because the transition to parenthood is already accompanied by numerous obstacles, including changes in psychological functioning, pregnant women may be at a higher risk of developing mental health problems during the pandemic. Pregnant women were considered to be more vulnerable to uneasy feelings. They are dealing with a variety of issues and stress, including constant nausea, exhaustion, and regular aches (7). Approximately 84% of women will experience stress throughout the antennal period of childbirth as a result of prior medical issues and sociocultural influences (8). Furthermore, most unfavorable life changes during pregnancy, such as financial difficulties, the death of a close family member, or worldwide events like the COVID-19 pandemic, can cause stress in the mother. Fear of contracting coronavirus and increased pressure to avoid negative health consequences for oneself or one’s children can have a variety of negative consequences for mothers and children, including obstetric complications (9), lower birth weight (10), child development delays (11), postpartum depression and other mental health risks (12) all of which can lead to poor child outcomes (13). Therefore, it is necessary to actively pay close attention to pregnant women’s mental health throughout the outbreak.

Additionally, during the pandemic, pregnant women are more likely to have sleep issues. According to many studies, approximately one-third of pregnant women in the United States reported receiving less sleep during the COVID-19 epidemic (14). On the other hand, any change in a pregnant woman’s sleep quality may have an impact on her feelings about labor pains and taking on the maternal role. In addition, sleep deprivation is connected to negative maternal outcomes such as daily dysfunction, weariness, and lower psychological relaxation among pregnant women (15). It may also have a role in the initiation, aggravation, and recurrence of mood disorders (16). Given the detrimental consequences of poor sleep quality, identifying the causes of sleep disruption is critical, with the objective of improving sleep quality in expectant mother as a possible therapy goal.

Pregnant women were more vulnerable to stress than women who were not pregnant. The lack of access to expected prenatal care as a result of the lockdown policy might further exacerbate the problem. A high degree of perceived stress has been associated to a higher risk of unfavorable cardiac outcomes and a higher likelihood of cardiac-related death (17). Furthermore, Ko et al. proposed that sleep quality was influenced by perceived stress (18, 19). In a negative sense, the sleep quality of pregnant women might be linked to the stress level they obtained (20), which could be jeopardized by the looming infectious illness pandemic in response (21, 22). Based on an animal research, long-term stress exposure promotes undesired sleep structural alterations, including reduced slow-wave sleep and increased rapid eye movement (REM) sleep (23). Sleep quality is negatively influenced by a person’s history of exposure to stressful life events, according to cross-sectional findings–both subjectively reported and objectively evaluated (24). For example, job stress might considerably increase the probability of experiencing irregular sleep disruption (25). To further, the deleterious effects of prenatal mother stress on poor sleep quality may be connected to an increased cortisol waking response and an overactive hypothalamus-pituitary-adrenal (HPA) axis (26, 27), all of which can have a variety of negative effects on sleep (28).

Resilience has been investigated as a possible moderator of the effect of stress exposure and negative consequence. People who have a higher level of resilience have a better psychological adjustment (29). In view of Rutter’s proposed protective mechanism for psychological resilience, resilience should serve a protective role in reducing undesirable chain reactions (30). Resilience has been shown to protect against stress during prior viral pandemics. It was discovered that resilient individuals exhibited lower levels of SARS-related concern than those who did not survive the pandemic (31). Contrary to popular belief, resilience has a positive link with sleep quality. According to a recent study, resilience training helps medical students manage occupational stress throughout their clinical year (32). Among those who scored high on resilience had a decreased chance of displaying poor sleep quality or using sleep medicines in the past month among HIV-positive people (33). As a result, it is suggested that resilience should be a crucial adjustment element for sleep quality even under stressful settings.

Amongst the small number of studies concerning the psychological outcomes of the lockdown policy during COVID-19, research on the association between perceived stress and sleep quality among pregnant women in China has not been conducted following the outbreak of COVID-19. The goal of this study was to see how perceived stress and resilience of pregnant women affected sleep quality when they were suffering from the COVID-19 pandemic. We further hypothesized that resilience during COVID-19 may moderate the association between stress and sleep quality, which might point to future avenues for psychological healthcare interventions during similar public health emergencies.

## Materials and Methods

### Settings and Study Population

Between March 30th and April 26th, 2020, a cross-sectional survey was conducted utilizing a multi-stage sampling approach to recruit participants. For the first stage, Wuhan (Hubei Province’s capital), Beijing, and Lanzhou (Gansu Province’s capital) were chosen for the following reasons. To begin, participants from Wuhan City and another area might represent pregnant women who are more and less affected by the lockdown policy in this situation, respectively. Second, Hubei (Central China), Beijing (North China), and Gansu (Western China) are not contiguous, therefore the province-wide lockdown measures in Hubei will not have a spillover impact. We chose a regional mother and child health care facility in each Chinese city in the second round. The underlying reason is that professional mother and child health care services are concentrated in each city’s regional hubs. Meanwhile, the income-related discrepancy in the healthcare-seeking process was generally overlooked by more than 95% of healthcare insurance coverage (34). As a result, regional clinics are the first choice for most women to seek prenatal care. We picked a regional mother and child health care center in each location to collect data from scattered populations. Convenience sampling was used in the third stage to enroll individuals from these two sites. We gave healthcare providers in charge of prenatal checkups at the study locations a QR code that led to an online questionnaire. When eligible pregnant women came in for their prenatal checkups, these healthcare providers offered them to participate in the study. Over 90% of Asian women had a gestation duration of fewer than 41 weeks, according to reports (35), which implies that the majority of Chinese women having a gestation period of more than 40 weeks would be those who were hospitalized and awaiting birth in China’s context. Women who were up to 40 weeks pregnant or less were included (similar inclusion criteria were used by Özkan et al.) (36); and resided in the local community throughout the COVID-19 pandemic, since the goal of this study was to look into pregnant women’s experiences at home instead of in a hospital facility. People with a history of mental illnesses were not allowed to participate. In this study, the gestational weeks of pregnant women ranged from 1 to 40 weeks, with an average gestational week of 26.19 weeks. 98% pregnant women are partnered, 2% pregnant women without partner. In line with the Declaration of Helsinki, the Ethics Committee for Scientific Research of the Chinese Academy of Sciences’ Institute of Psychology granted ethical permission, permission number was H20003. All subjects gave written informed permission to participate in the study during the final recruitment.

### Measures

#### Insomnia Severity Index

The Insomnia Severity Index (ISI) is an instrument widely used to assess the severity of insomnia symptoms. The ISI has demonstrated good reliability with a Cronbach’s alpha of 0.9 (37). In the present study, the Cronbach’s alpha was 0.93. The scale has been used to assess pregnant women’s subjective sleep quality (38). Furthermore, compared to other scales, this one has fewer components and is more convenient. Scores vary from 0 to 28 on the ISI worldwide scale. A higher score indicates a poorer quality of sleep. A total score of 0∼7 means “no insomnia symptoms,” while a total score of 8∼14 means “mild insomnia symptoms,” the overall score of 15∼21 means “moderate insomnia symptoms,” 22∼28 means “severe insomnia symptoms.”

### Perceived Stress Scale

The Perceived Stress Scale (PSS) is the most often used psychological tool for assessing stress perception. It is a metric for how stressful certain situations in one’s life are regarded (39). The PSS was graded on a 5-point Likert scale ranging from not at all (0) to highly (4) depending on how often they occurred in the month leading up to the survey and is intended to capture how unpredictable and unmanageable respondents’ lives are. The PSS scores are calculated by inverting the scores on four positive items, such as 0 = 4, 1 = 3, and 2 = 2. The overall score was then computed. The higher the score on the scale, the higher the subject’s stress level. The lower the score on the scale, the less stressed the participants are. This scale has high validity and reliability, according to studies from several countries (40, 41). Cronbach alpha evaluated the scale’s internal consistency at 0.85 (42). Cronbach’s alpha was 0.72 in this research.

#### Resilience Scale

Resilience is the ability to recover from adversity, conflict, failure, and even positive events (43). The Connor and Davidson Resilience Scale (CD-RISC) (44)was used to assess resilience (including tenacity, strength, and optimism), which measures personal attributes that enable people to flourish despite being exposed to stress and trauma. The Connor-Davidson Resilience (CD-RISC-10), a 10-item measure derived from a 25-item scale. Each item is assessed on a 5-point Likert-type scale (from 0 to 4), with higher scores suggesting a higher level of resilience. The CD-reliability RISC-10‘s and validity are further demonstrated by its extensive use in a Chinese population (45). In this study, the Cronbach’s alpha of the CD-RISC was 0.93.

#### Covariates

A total of 2,115 people were recruited in this study. Structured questionnaires were utilized by trained research workers to gather social-economic data as well as lifestyle information and other specific characteristics. Age, level of education (high school/college/undergraduate/post-graduate), annual household income (RMB 80,000/80,000−300,000/> 300,000), financial loss during COVID-19 (no financial loss/20,000/20,000−49,999/50,000), and whether they were infected with COVID-19 alone or in relatives and friends were all socioeconomic status variables. History of physical disease, mental diagnosis, drug use, smoking (never smoking/already stopped smoking/continuing smoking), and drinking behaviors (never drink: never drink alcohol in life/already quit drinking) were among the health behavior factors. The number of births, vomiting during pregnancy, daily monitoring of the fetus (Pregnant women answered these questions by recalling their daily attention to fetal movement during pregnancy and based on the actual situation), abdominal pain during pregnancy, pregnancy’s influence on mobility (meaning that pregnancy may cause difficulty going to different locations), worries and fears about childbirth, and car accidents were all pregnancy-related variables.

In this study, smokers were defined as adults who had smoked 100 cigarettes in their lifetime and currently smoke cigarettes every day (daily) or some days (nondaily). Alcohol users were characterized as people who drank more than five drinks on a regular basis (rather than just sometimes). The occasional, light, and infrequent users were excluded from the study.

### Statistical Analysis

Using SPSS version 25.0, descriptive analysis, correlation analysis, Mann–Whitney tests, and Kruskal–Wallis tests were performed after data collection. PROCESS version 3.5 was used to create the moderating model.

Harman’s single-factor test was used to assess the common method variance in this study. The findings revealed that no one factor could account for the bulk of variation (the maximum component only explained 36.57 % of total variance), indicating that there was no common technique bias in this research. All results in this study indicated univariate non-normality for all measured variables. The variations in sleep quality, resilience, and perceived stress were tested using Mann–Whitney and Kruskal–Wallis tests in relation to categorical socio-demographic factors. The direction and magnitude of the correlations between perceived stress, resilience, and insomnia severity were also determined using Pearson Perceived stress was considered as the independent variable. Insomnia Severity was considered as the dependent variable. To see if resilience mitigated the connection between perceived stress and insomnia severity, researchers used SPSS 25.0 to run multiple linear regressions. We used simple slope analyses to compute the strength of the link between perceived stress and insomnia symptoms scores with high (1 SD above the mean) and low (1 SD below the mean) levels of resilience scale scores to assist interpret the interaction. To decrease multicollinearity, all continuous variables were centered before the analysis, and the interaction term was calculated using the centered variables.

## Results

### Participants’ Demographic Characteristics

In total, 2115 Chinese pregnant women were investigated. Education, annual household income, financial loss during COVID-19, whether they are afflicted with COVID-19, and/or whether they have COVID-19 family and acquaintances, smoking, drinking, number of births, vomiting during pregnancy, daily monitoring of the fetal abdominal discomfort, pregnancy’s impact on mobility, anxiety and fears about birthing, and caregiver status are among the general demographic information included in the study. Table 1 shows the correlation between Insomnia Severity and these variables.

**TABLE 1:** Demographic status of the sample.

Variables (*n* = 2115)	Insomnia Severity		Resilience		Perceived Stress	
	Z/t	*p*	Z/t	*p*	Z/t	*p*
Age (years)	0.002	0.928	0.034	0.114	−0.023	0.289
From Wuhan (Yes = 0, No = 1)	−6.157	< 0.001	−3.742	< 0.001	−3.431	0.001
Drinking	11.350	0.003	5.180	0.075	2.097	0.350
Smoking	10.095	0.006	0.668	0.716	5.058	0.080
Nausea and vomiting during pregnancy	20.825	< 0.001	15.161	0.002	18.866	< 0.001
Daily attention to fetal movement	−2.857	0.004	−1.896	0.058	−2.972	0.003
Impact of pregnancy on action	141.740	< 0.001	26.881	< 0.001	22.471	< 0.001
Be taken care of	1.428	0.490	15.145	0.001	50.589	< 0.001
Any worries or fears about childbirth	−10.600	< 0.001	−7.117	< 0.001	−8.415	< 0.001
Degree of Education	6.440	0.092	63.549	< 0.001	85.410	< 0.001
First Child	−3.216	0.001	−3.283	0.001	−3.071	0.002
Annual Household Income	1.043	0.594	49.368	< 0.001	109.648	< 0.001
Financial Loss in COVID-19 (RMB)	40.047	< 0.001	27.326	< 0.001	32.138	< 0.001
Stomach ache	25.120	< 0.001	5.468	0.141	1.847	0.605
Relatives or friends are infected with covid-19	−0.292	0.771	−0.070	0.944	−0.257	0.797

*The p-values were tested using the Pearson Correlation, Mann–Whitney tests, and Kruskal–Wallis tests.*

During the COVID-19 pandemic, according to the present study 18.53% of 2,115 pregnant women are having sleeplessness,. The participants were 30.52 years old on average (*SD* = 9.67, range 19–47). 55.00% of the respondents had at least a bachelor’s degree and 31.35% had a low average yearly household income (≤80,000 RMB), and 84.92% were primiparas. 2% pregnant women were single mothers and 98% with partner. The majority of those who were affected by the lockdown policy did not drink alcohol or smoke. Furthermore, the majority of pregnant women surveyed reported nausea and vomiting, stomachaches, daily observed fetal activity, were cared for, and had anxieties or anxiety about delivering. In general, those who were in Wuhan had already stopped drinking and smoking, suffered from nausea and vomiting during pregnancy, had daily fetal movement monitored, were concerned about childbirth, expected to have their first child, resulted in a greater financial loss, and had stomachache had a larger chance of experiencing insomnia symptoms than their peers. Furthermore, those who were in Wuhan and suffered from nausea and vomiting during pregnancy, were impacted by pregnancy on action, were not taken care of, were concerned about childbirth, had less education, were expecting to have their first child, had less annual household income, and had more financial loss as a result of COVID-19 were more likely to have lower resilience scores than their counterparts. Finally, those in Wuhan who suffered from nausea, vomiting, and stomachaches during pregnancy, did not daily monitor fetal movement, were impacted by pregnancy on action, were not taken care of, were concerned about childbirth, had less education, expected to have their first child, had less annual household income, and had more financial loss due to COVID-19 had a higher risk of perceiving stress than their counterparts.

### The Correlation Relationship Between Perceived Stress, Resilience, and Insomnia Severity

Table 2 shows a Pearson correlation analysis of perceived stress, resilience, and insomnia severity. It was discovered that resilience scores were negatively correlated with perceived stress (*r* = −0.470, *p* < 0.001) and insomnia severity (*r* = −0.270, *p* < 0.001), while perceived stress was positively correlated with insomnia severity (*r* = 0.357, *p* < 0.001), indicating that higher perceived stress was associated with lower resilience and worse sleep quality (higher insomnia severity), whereas higher resilience was associated with better sleep quality (lower insomnia severity).

**TABLE 2:** Correlations among study variables (*N* = 2115).

Variables	Mean	SD	1	2	3
1. Perceived Stress	13.6*0*	5.69	–	–	–
2. Resilience	29.9*0*	7.84	−0.469**	–	–
3. Insomnia Severity	4.39	4.53	0.360**	−0.272**	—

*p < 0.05, **p < 0.01, ***p < 0.001.

The intensity of insomnia is related to perceived stress and resilience. Relationships between perceived stress and insomnia severity mediated by resilience are represented by regression lines (1 *SD* above and below the mean, two-way interaction). Slopes of low resilience (β=0.337, *p* < 0.001) and high resilience (β=0.1392, *p* < 0.001) are both significant.

### Moderating Effects

Regression analyses were used to see if resilience might mitigate the negative consequences of perceived stress. Table 3 shows the results of the regression analysis. To control the effect on the variables and to increase the overall R^2^ to increase the power of the statistical test, Drinking, Smoking, Nausea and vomiting during pregnancy, Daily attention to fetal movement, impact of pregnancy on action, Be taken care of, Any worries or fears about childbirth, Degree of education, First Child, Annual household income, Financial loss in COVID-19 and Stomachache were controlled as covariates in the regression analysis. Perceived stress was found to be a positive predictor of insomnia severity in Table 3. The interaction term between perceived stress and insomnia severity was found to be significant, suggesting that the relationship between perceived stress and insomnia severity varied depending on resilience.

**TABLE 3:** Regression analysis examining the role of Perceived Stress in predicting Insomnia Severity.

Insomnia Severity	β	SE	Z	Two-tailed *p*-value
**Covariates**				
Drinking	−0.345	0.284	−1.215	0.225
Smoking	0.246	0.225	1.090	0.276
Nausea and vomiting during pregnancy	0.255	0.150	1.706	0.088
Daily attention to fetal movement	0.716	0.230	3.113	0.002
Impact of pregnancy on action	1.264	0.167	7.562	*p* < 0.001
Be taken care of	0.117	0.174	0.670	0.503
Any worries or fears about childbirth	0.900	0.194	4.637	*p* < 0.001
Degree of education	0.100	0.110	0.868	0.386
First Child	−0.425	0.247	−1.720	0.086
Annual household income	0.214	0.154	1.390	0.165
Financial loss in COVID-19	0.226	0.077	2.924	0.004
Stomachache Predictors	0.467	0.154	3.025	0.003
Perceived Stress	0.238	0.0183	13.000	*p* < 0.001
Resilience	−0.044	0.0315	−3.286	0.001
**Interaction**				
Perceived Stress and Resilience	−0.013	0.002	−6.034	*p* < 0.001

**FIGURE 1:**
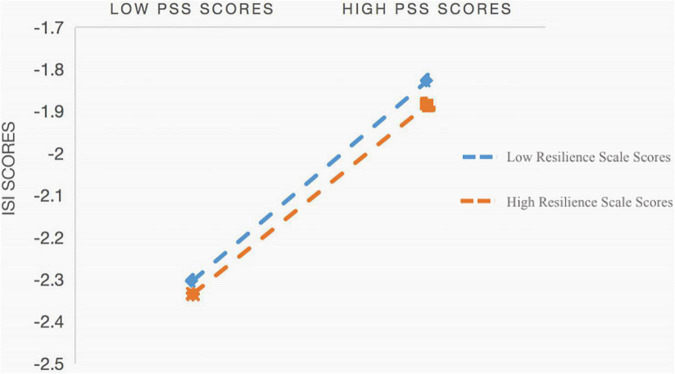
Interaction of Perceived Stress Scores and Resilience Scores on Insomnia Severity Scores. PSS means Perceived Stress Scale, ISI means Insomnia Severity Scale.

We displayed the relationship between perceived stress (1 *SD* above or below the mean) and insomnia severity at different degrees of resilience to better understand the nature of the interaction (Figure 1). According to simple slopes testing, the association between perceived stress and insomnia intensity was statistically significant at various levels of resilience. The interaction between perceived stress and resilience was significant and negative on insomnia severity in the moderation model (β= −0.126, *p* < 0.001), implying that resilience mitigated the relationships between perceived stress and insomnia severity. Subgroup analysis conducted for the moderation of perceived stress and insomnia severity indicated that their effects differentiated the high resilience and low level of resilience. Pregnant women with low levels of resilience and high levels of perceived stress predicted higher insomnia severity (β= 0.337, *p* < 0.001). Pregnant women with high levels of resilience and high perceived stress also predicted higher insomnia severity (β= 0.1392, *p* < 0.001). Furthermore, the effect of perceived stress-related insomnia severity was less pronounced in the high resilience group than in the poor resilience subgroup.

## Discussion

The present study used hierarchical regression analysis to confirm the moderating impact of resilience on the relationship between perceived stress and sleep quality in Chinese pregnant women during COVID-19. Sleep quality, in particular, was found to be inversely related to stress perception. Furthermore, 18.53% of the individuals in this research reported having insomnia. Contrary to prior literature, the figure, 18.53%, is explicitly smaller compared to estimates of Chinese pregnant women prior to COVID-19, which was 49.4% (46). Table 1 also shows that women with nausea and vomiting were more likely to have poor sleep quality, replicating FitzGerald and Davis’ prior results that women with moderate/severe pregnancy-related nausea and vomiting were more likely to have sleep disturbance than those with none/mild sickness (47, 48). Such findings might be explained that the lockdown strategy enhanced pregnant women’s resilience, therefore reducing the impact of perceived stress on sleep quality. To elaborate, family bonding might be reinforced even more when family members spent more time together, supported one another, and faced hardships together. Meanwhile, for pregnant women, their partners’ mental health, the availability of social networks, and the companionship of family and friends all contribute to psychological resilience and emotional stress reduction.

During COVID-19, there was also a substantial negative association between felt stress and sleep quality, according to the current study. These findings back up previous findings from a study with pregnant women. Since the COVID-19 outbreak in December 2019, researchers have documented the pandemic’s negative impacts on mental health, as well as the anticipated complicated impact on pregnant women. Hayase observed that in pregnant women, shorter sleep duration and increasingly worse sleep quality were linked to increased subjective stress (45). According to Carney et al. worrisome thoughts might prevent people from going asleep due to a lack of relaxation, interfering with circadian rhythms (49). Harvey also indicated that during the pre-sleep phase, stress enhanced cognitive and somatic arousal, affecting total sleep quality (50). According to the previous studies, a decrease in perceived stress was related to a significant improvement in sleep quality (51), this suggests that controlling and reducing prenatal mother stress might be an effective way to improve sleep quality.

The outcomes of the present study confirmed the moderation effect of resilience on perceived stress and insomnia symptoms. It indicated that increased resilience was significantly associated with improved sleep quality in pregnant women during COVID-19, which was consistent with previous research (52). The findings revealed that the association between stress and insomnia symptoms is less for persons with high levels of resilience than for those with low levels of resilience. To illustrate, individuals with high resilience have favorable characteristics (e.g., high cope self-efficacy, optimistic emotions, realistic optimism, and cognitive flexibility) that might enable them to positively get used to and keep good sleep quality when they are confronted with acute or chronic stress (53). The moderation impact of resilience on stress and sleep quality demonstrated that pregnant women with more perceived stress and less resilience, subsequently, had poorer sleep quality. In contrast, pregnant women with less perceived stress and greater resilience, subsequently, had more favorable sleep quality. Because of the severe pandemic in Wuhan, individuals in Wuhan felt more stressed and had less resilience than those in other places, as seen in Table 1. As a result, individuals had increased sleeplessness symptoms. The current findings are consistent with a previous study, which found that the severity of perceived stress was negatively correlated with resilience, which was associated to psychological and physical health, including sleep quality. This finding is consistent with prior research indicating resilience is a protective feature that aids people in adapting to poor environmental quality (54). The existing literature has attached sleep quality with psychological resilience from a neurobiological perspective (55, 56). One potential explanation is that high resilience could sustain the HPA axis at an optimum level of activation; that is, high enough to get adjusted to danger but not so high as to trigger superfluous fear, anxiety, and depression, thus enabling the resilient individual to prevent psychosomatic disorders like sleep disturbance (57).

Further research is needed in this area in the future to better understand the molecular and psychological mechanisms. Resilience’s moderating effect on perceived stress and sleep quality provides fresh insight into the components that influence sleep quality. Resilience, in particular, might be considered a component to be addressed in sleep quality enhancement programs for pregnant women (58, 59). On the other hand, local community and government agencies should provide more psychological service to individuals to cope with their stress. Pregnant women would feel more secure, and additional psychological support measures might help them feel less worried. As a result, even if they have a low degree of resilience, they may be able to have decent sleep.

## Limitation

There are certain limitations to this study that should be mentioned. For starters, the cross-sectional design made study difficult to confirm the causal link between resilience, stress, and insomnia symptoms. To determine the causal influence of perceived stress on insomnia symptoms during pregnancy, longitudinal studies are required. Second, the current study only used self-report ratings, which might contribute to methodological variability (CMV). More study using a variety of approaches to measure sleep quality is needed. Third, We did not analyze the influence of the presence of partners on the mental health of pregnant women in this study because the majority of the pregnant women polled were pregnant women with partners. Future research should consider the crucial function of partners in our transition from individual to parent.

Nonetheless, there were a few positive aspects to this research. In COVID-19 pregnant women in China, this was the first study to indicate that resilience moderates the association between perceived stress and sleep quality, and the sample size was large: 2115 pregnant women were studied. These findings contribute to a better understanding of the relationship between resilience, perceived stress, and insomnia symptoms in pregnant women, and offer a new direction to develop interventions to advance sleep quality.

## Data Availability Statement

The raw data supporting the conclusions of this article will be made available by the authors, without undue reservation.

## Ethics Statement

The studies involving human participants were reviewed and approved by the Ethics Committee for Scientific Research of the Chinese Academy of Sciences’ Institute of Psychology. The patients/participants provided their written informed consent to participate in this study.

## Author Contributions

HZ and ZT were responsible for writing-original draft preparation, reviewing and editing, methodology, and analyzed the data. YZ, ZZ, and LL were responsible for statistical analysis and manuscript revision. CZ, JY, and YW were responsible for data acquirement. WH and JW were responsible for study design and supervision. All authors have given final approval for its publication.

## Conflict of Interest

The authors declare that the research was conducted in the absence of any commercial or financial relationships that could be construed as a potential conflict of interest.

## Publisher’s Note

All claims expressed in this article are solely those of the authors and do not necessarily represent those of their affiliated organizations, or those of the publisher, the editors and the reviewers. Any product that may be evaluated in this article, or claim that may be made by its manufacturer, is not guaranteed or endorsed by the publisher.
